# Cell-Penetrating Peptide–Peptide Nucleic Acid Conjugates as a Tool for Protein Functional Elucidation in the Native Bacterium

**DOI:** 10.3390/molecules27248944

**Published:** 2022-12-15

**Authors:** Yasuhito Yokoi, Yugo Kawabuchi, Abdullah Adham Zulmajdi, Reiji Tanaka, Toshiyuki Shibata, Takahiro Muraoka, Tetsushi Mori

**Affiliations:** 1 Department of Biotechnology and Life Science, Tokyo University of Agriculture and Technology, 2-24-16 Naka-cho, Koganei-shi 184-8588, Tokyo, Japan; 2 Department of Life Sciences, Graduate School of Bioresources, Mie University, 1577 Kurima-machiya-cho, Tsu-shi 514-8507, Mie, Japan; 3 Department of Applied Chemistry, Graduate School of Engineering, Tokyo University of Agriculture and Technology, 2-24-16 Naka-cho, Koganei-shi 184-8588, Tokyo, Japan

**Keywords:** cell-penetrating peptides, peptide nucleic acid, uncharacterized proteins, native bacterium

## Abstract

Approximately 30% or more of the total proteins annotated from sequenced bacteria genomes are annotated as hypothetical or uncharacterized proteins. However, elucidation on the function of these proteins is hindered by the lack of simple and rapid screening methods, particularly with novel or hard-to-transform bacteria. In this report, we employed cell-penetrating peptide (CPP) –peptide nucleotide acid (PNA) conjugates to elucidate the function of such uncharacterized proteins in vivo within the native bacterium. *Paenibacillus*, a hard-to-transform bacterial genus, was used as a model. Two hypothetical genes showing amino acid sequence similarity to ι-carrageenases, termed *cgi*A and *cgi*B, were identified from the draft genome of *Paenibacillus* sp. strain YYML68, and CPP–PNA probes targeting the mRNA of the acyl carrier protein gene, *acp*P, and the two ι-carrageenase candidate genes were synthesized. Upon direct incubation of CPP–PNA targeting the mRNA of the *acp*P gene, we successfully observed growth inhibition of strain YYML68 in a concentration-dependent manner. Similarly, both the function of the candidate ι-carrageenases were also inhibited using our CPP–PNA probes allowing for the confirmation and characterization of these hypothetical proteins. In summary, we believe that CPP–PNA conjugates can serve as a simple and efficient alternative approach to characterize proteins in the native bacterium.

## 1. Introduction

Proper and correct functional analyses of newly discovered proteins is crucial in order to elucidate the role of the protein in a given metabolic reaction or cellular system. Thus far, elucidation of such novel proteins has depended highly on heterologous protein expression techniques whereby the gene encoding for the protein is cloned into specified cloning vectors, allowing for the protein to be overexpressed in easily cultivable and manipulative host strains [1]. Well-established with numerous commercially available protein expression system lineups [2,3,4], many newly discovered proteins can be easily and efficiently characterized. However, the expression of the target protein using this approach still requires validation and optimization based on factors such as the physiological role and solubility of the expressed protein, the presence or absence of post-transcriptional modification, and codon usage. Furthermore, protein expression can be hindered by microbial host compatibility if the target protein originates from unknown or uncultivable strains [5], while some hosts are also known to rearrange genes or alter them [6] as a defense mechanism to prevent cytotoxic effects of the foreign protein to itself. To counter this effect, efforts to establish new microbial expression hosts are being conducted, however, this process is tedious, time consuming, and still does not guarantee the expression of the target gene or gene clusters.

Due to the difficulties faced by conventional heterologous protein expression, alternatively, researchers have taken advantage of the vast amount of data generated by next generation sequencers from online databases to perform protein functional analyses. Gene annotation can now be further facilitated by in silico algorithms such as structural and functional gene annotation, where it is possible to make predictions and speculate the biological characteristics of new or candidate proteins based on orthology, conserved domains, subcellular targeting signals or its tertiary structure [7,8]. In addition, this approach is applicable in predicting the function of novel proteins attained from uncultivable hosts or those that were unsuccessfully expressed in current conventional protein expression systems. However, despite the availability and applicability of these in silico approaches, a downside to their application is the possibility of incorrect protein characterization [9,10,11]. Proteins whose functions have been predicted based on a low number of available samples, or proteins that have not been experimentally justified, can be used as a base for prediction. Today, even with the availability of high-throughput sequencing and computationally advanced protein prediction tools, we still face problems with misannotated proteins in public databases [12].

With the challenges faced from in vitro approaches, such as the current heterologous expression and in silico prediction systems, efforts to further promote more effective and accurate characterization of proteins have been directed towards enhancing in vivo expression of the proteins within the native bacterium. One approach is the alteration of the cultivation environment of the bacterium by employing extreme environmental factors [13]. Altering the cultivation environment by applying different stresses results in the microbe expressing more of the target protein or utilizing a different metabolic pathway to counter the stress applied, leading to the expression of “silent” or “dormant” proteins. Biosynthetic engineering approaches, such as the recombination of transcription regulators via gene refactoring, have also been introduced to promote the expression of “silent” gene clusters of microbial natural products [14,15,16]. Such an approach allows the identification of modification enzymes that can be employed as key components for the synthesis of novel lead compounds. Although the implementation of such approaches is effective and has allowed for more proteins to be discovered, validating the function of the protein of interest within the native bacterium is challenging. Researchers still need to resort to current conventional methods in order to functionally characterize the target protein.

Our group focuses on cell-penetrating peptides (CPPs) as a tool to promote the delivery of biomolecules into bacteria, where we have been successful in promoting CPP permeation in diverse bacterial strains by optimizing the abiotic factors [17] and using unnatural amino acid residues [18]. In this study, we challenged the use of CPP in combination with peptide nucleic acid (PNA) to elucidate the function of proteins in vivo within the native bacterium, taking it one step further by testing the approach with a hard-to-transform bacterium, *Paenibacillus*, as a model. We believe that our work will allow for a more simple and efficient approach to characterizing proteins, eventually working towards a new standard approach in protein functional analysis.

## 2. Materials and Methods

### 2.1. Genomic DNA Extraction

*Paenibacillus* sp. YYML68 was cultured in Marine Broth 2216 (MB; BD Difco, Tokyo, Japan) at 30 °C for 2 days with agitation. The cultured cells were collected via centrifugation at 7500× *g*, 10 min, and genomic DNA extraction was performed using the Genomic DNA Buffer Set and Genomic-tip 100/G (QIAGEN, Tokyo, Japan) based on the manufacturer’s protocols. The extracted DNA concentration was quantified using the Qubit dsDNA HS Assay Kit with a Qubit 3.0 fluorometer (Thermo Fischer Scientific, Tokyo, Japan).

### 2.2. Genome Sequencing and Assembly

Genomic DNA sequencing was performed using the Oxford Nanopore Technologies (ONT; Oxford, UK) and Illumina (Tokyo, Japan) platforms. For ONT sequencing, a genomic library was prepared using the Rapid sequencing kit (SQK-RAD004) based on the manufacturer’s protocols. Sequencing was performed using a MinION system with a run time of 48 h, using a R9.4 flow cell. For Illumina sequencing, library preparation and sequencing were performed by Macrogen Japan Corporation (Tokyo, Japan). A library was prepared using the TruSeq DNA PCR-Free kit from 1 µg of genomic DNA, followed by a 151 bp paired-end sequencing run using a NovaSeq6000 system.

To assemble the attained reads, basecalling of ONT raw data reads was initiated using Guppy v3.4.5 (ONT). Sequence adapters were removed from the obtained sequence reads using Porechop v0.2.4 (https://github.com/rrwick/Porechop, accessed on 12 May 2022) and sequences with Phred Scores (Q) below 8 or below 1000 bp in length were removed using NanoFilt v2.5.0 (https://github.com/wdecoster/nanofilt; [19], accessed on 12 May 2022). Final contigs were subsequently generated via de novo assembly using Flye v2.7 (https://github.com/fenderglass/Flye; [20], accessed on 12 May 2022) using the remaining reads. Subsequently, the contigs attained from the ONT sequencing were corrected using Pilon v1.2.3 (https://github.com/broadinstitute/pilon; [21], accessed on 12 May 2022) with the reads attained from Illumina sequencing. Adapters from the Illumina sequencing reads used for correction were priorly processed with Trimmomatic v0.36 (https://github.com/usadellab/Trimmomatic; [22], accessed on 12 May 2022), accordingly. The resulting contig was used as the draft genome for strain YYML68. Genome completeness of the final draft genome was evaluated based on the conservation of single-copy orthologous genes using BUSCO v4.0 (https://busco.ezlab.org; [23], accessed on 12 May 2022).

### 2.3. Gene Annotation and Identification of Carrageenase Encoding Genes

Genes in the final draft genome of strain YYML68 were annotated using the web tool Rapid Annotation using Subsystems Technology (RAST; http://rast.nmpdr.org/, accessed on 12 May 2022) [24]. Protein sequences annotated by RAST were extracted and carbohydrate-active enzymes (CAZymes) were identified using the dbCAN2 meta server ((http://bcb.unl.edu/dbCAN2/, accessed on 12 May 2022) [25] based on the following tools and criteria: HMMER (E-Value < 1 × 10^−15^, coverage > 0.35), DIAMOND (E-Value < 1 × 10^−12^) and Hotpep (Frequency > 2.6, Hits > 6). Using the annotation results from RAST and dbCAN2, κ-carrageenase (CAZy family GH16), ι-carrageenase (CAZy family GH82) and λ-carrageenase (CAZy family GH150) encoding candidate genes were identified. Finally, the protein sequences of the candidate carrageenases were aligned with all the sequences from the designated GH family, and a phylogenetic tree was generated using the Neighbor-Joining method without an outgroup using Geneious Prime v2020.2.5 (https://www.geneious.com, accessed on 12 May 2022).

### 2.4. Carrageenan Degradation by Paenibacillus sp. YYML68

*Paenibacillus* sp. strain YYML68 was cultured, collected as described above, and washed twice with filter-sterilized seawater. Upon washing, the cells were counted using a bacteria-counting chamber (Erma Inc., Tokyo, Japan) and were inoculated to 3 mL (final concentration of 1.0 × 10^5^ cells/mL) of 4× diluted MB supplemented with 1% ι-carrageenan, 1% κ-carrageenan or 2% λ-carrageenan, respectively, prepared in 15 mL centrifugation tubes. Each sample was incubated at 30 °C with gentle agitation, and degradation of each carrageenan type was measured at 0, 8, 16 and 24 h. Non-bacteria inoculated MB-carrageenan samples were used as negative controls. Taking advantage of the fact that viscous carrageenan solutions lose their viscosity due to enzymatic degradation, carrageenan degradation was determined simply by measuring the increase in height of the liquid level of the respective cultures upon stirring, and comparing it to the negative controls at the designated time points. Stirring of the samples was performed using a Vortex-Genie 2 Mixer (Scientific Industries, New York, USA) at the lowest speed setting.

#### 2.4.1. CPP–PNA Design and Synthesis

**Figure 1:**
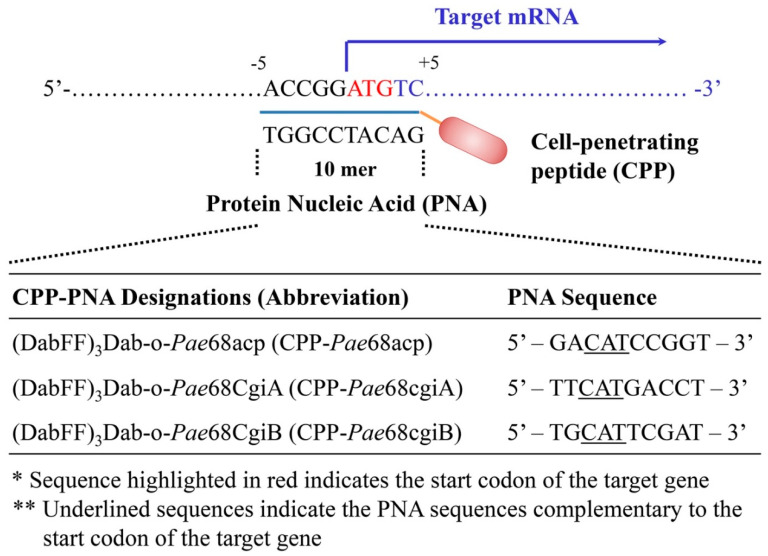
RNA translation inhibition with CPP-PNA and the CPP-PNA probes used in this work. Dab and F each represents the amino acid residues 2,4-diaminobutyric acid and phenylalanine that make up the CPP. 2-aminoethoxy-2-ethoxy acetic acid (AEEA) represented here as the -o- linker is used to link CPP with PNA. All probes were designed and synthesized in-house.

The CPP–PNA probe design and sequences used in this work are provided in Figure 1. Briefly, the CPP sequence, (DabFF)_3_Dab, used in this work comprised a combination of an unnatural cationic amino acid (2,4-diaminobutyric acid, Dab) and phenylalanine (F), arranged similarly to that in our previous reports [17]. 2-aminoethoxy-2-ethoxy acetic acid (AEEA) was used as the linker between (DabFF)_3_Dab and PNA. The PNA sequences, for the designated CPP-*Pae*68acp, CPP-*Pae*68cgiA and CPP-*Pae*68cgiB probes, were designed to bind to the start codon of the mRNA encoding for the acyl carrier protein, AcpP, the candidate ι-carrageenase, CgiA, and the candidate ι-carrageenase, CgiB, respectively. All CPP–PNA probes were synthesized using standard peptide solid-phase synthesis protocols, purified with a reversed-phase high-performance liquid chromatography (RP-HPLC) system (JASCO Corp., Tokyo, Japan), and subjected to mass spectrometry analysis using a MALDI-TOF MS (autoflex speed TOF) system (Bruker Japan K. K., Kanagawa, Japan) analysis for successful synthesis confirmation.

#### 2.4.2. Inhibition Assays Using CPP–PNA

To perform the growth inhibition assay, we used the CPP-*Pae*68acp probe. 3 mL MB inoculated with strain YYML68 to a final concentration of 1.0 × 10^5^ cells/mL were used as starter cultures. The CPP-*Pae*68acp probe was added to the starter cultures at final concentrations of 1, 2, 4, 6, 8 and 10 µM and incubated at 30 °C with agitation. Culture turbidity was measured at 0, 2, 4, 6, 8, 10, 12, 24 and 36 h, respectively, using an OD monitor miniphoto 518R (Taitec Corp., Saitama, Japan) at 660 nm. Non-CPP-*Pae*68acp probe inoculated starter cultures were used as negative controls.

To evaluate the function of the candidate carrageenases CgiA and CgiB within strain YYML68, four combinations using the CPP-*Pae*68cgiA and CPP-*Pae*68cgiB probes were tested; −/−, +/−, −/+ and +/+ (+ and − symbols represent the addition or non-addition of the probes, respectively). 3 mL 4× diluted MB supplemented with 1% ι-carrageenan, inoculated with strain YYML68 to a final concentration of 1.0 × 10^5^ cells/mL were used. The final concentration of the added probes was 8 µM. Each sample was incubated at 30 °C with gentle agitation, and degradation of ι-carrageenan, measured based on medium viscosity as described above, was performed at 0, 8, 16 and 24 h. Non-strain YYML68 inoculated medium and medium inoculated with a non-carrageenan degrading bacterium, *Bacillus megaterium* were used for comparison.

#### 2.4.3. Protein Structure Prediction and Comparative Analysis

Amino acid sequences of the candidate ι-carrageenases CgiA and CgiB were uploaded to the protein structure homology-modelling server SWISS-MODEL (https://swissmodel.expasy.org/interactive; [26], accessed on 12 May 2022) to attain predicted tertiary and secondary structures. Tertiary structure models were used for structural comparison against ι-carrageenase crystalized models, while the secondary structure sequences were used for homology comparison based on sequence similarity and ι-carrageenase conserved active residues. ι-carrageenase conserved active residues were determined based on previously reported ι-carrageenases of *Paenibacillus* sp. HB172198 (PaeCgi; QCT00926.1), *Microbulbifer thermotolerans* JAMB-A94^T^ (MicCgi; BAJ40863.1) and *Pseudoalteromonas atlantica* T6c (PseCgi; ABG39405.1) [27,28]. The presence of signal peptides within CgiA and CgiB was determined using SignalP v.6.0 (https://services.healthtech.dtu.dk/service.php?SignalP-6.0, accessed on 12 May 2022). The secondary structure sequence alignment and image was generated using Geneious Prime v2020.2.5.

### 2.5. Graph Presentation and Analysis

All graphs including *t*-test scores were generated using Prism9 v9.4.1 (GraphPad Software Inc., San Diego, CA, USA).

## 3. Results

### 3.1. Candidate Carrageenase Genes in Paenibacillus sp. YYML68

*Paenibacillus* sp. strain YYML68 is a bacterium that can efficiently degrade crude carrageenan (Figure S1). At the time of its isolation, in order to identify the genes encoding carrageenan-degrading enzymes or carrageenases, we sequenced the genome of this bacterium and annotated the genes. However, we were unable to identify any genes designated as carrageenases (Unpublished data). Since then, the project to isolate carrageenases from the bacterium was put on hiatus. Now that we can use CPP–PNA as a simple and efficient way to possibly identify the carrageenase-encoding candidate genes, strain YYML68 was used as the model bacterium for this work.

To refine the genome and to improve the probability of identifying possible candidate genes, we re-sequenced the genome of strain YYML68 using a combination of ONT and Illumina sequencing. Upon sequence assembly, we attained a single contig of 5,091,020 bp in size with GC contents of 53.5%. Analysis using BUSCO resulted in the identification of 444 single orthologue genes, accounting for 98.6% of the total 450 genes from the Bacillales order, suggesting that the sequenced genome was nearly complete. Gene annotation using RAST resulted in 4671 coding sequences (CDS) where 1022 genes (21.9%) were classified to 293 subsystems, while 1423 genes (30.5%) were uncategorized. On the other hand, 2226 genes (47.6%), nearly half of the total genes, were annotated as hypothetical proteins. Using this annotation result, although we did not identify any genes annotated as carrageenases, we successfully identified two candidate genes annotated as hypothetical proteins, HP60 and HP61, now termed as *cgi*A and *cgi*B, each encoding for the proteins CgiA and CgiB, that showed 67.3% and 76.8% amino acid sequence similarity to the ι-carrageenase identified from *Paenibacillus* sp. HB172198 (QCT00926.1) within the GH82 family (Figure S2). The amino acid sequence homology between CgiA and CgiB was 69.5%. The draft genome of *Paenibacillus* sp. YYML68 and the gene sequences of *cgi*A and *cgi*B has been deposited at DDBJ/EMBL/GenBank under the accession number BQYI01000001 (first version), LC730803 and LC730804, respectively.

**Figure 2:**
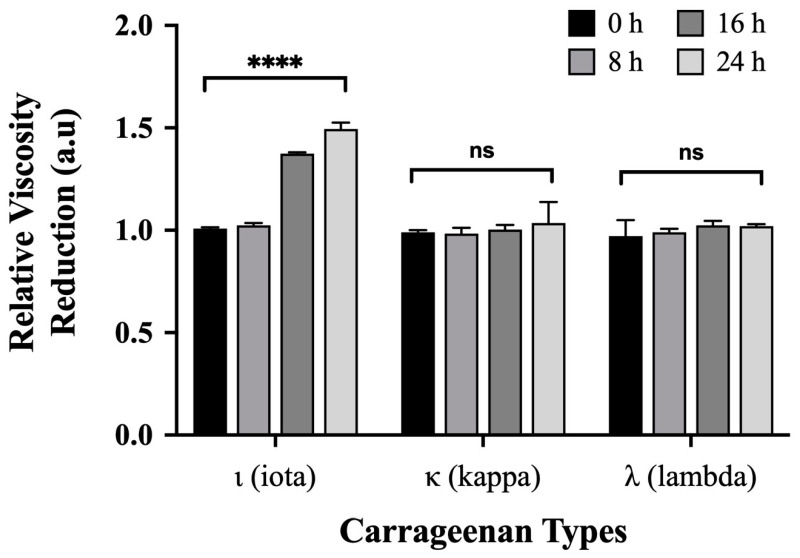
Degradation of ι, κ or λ-carrageenan using *Paenibacillus* strain YYML68. The concentration of the carrageenan types was 1%, 1%, and 2% respectively. Samples were measured at 0, 8, 16 and 24 h time points, and degradation was determined by the reduction in media viscosity in comparison to non-strain YYML68 treated samples. All samples were performed in triplicates. Statistical significance (*p* < 0.05) was determined by *t*-test where **** indicates the level of significance. ns: non-significant.

Simultaneously, we tested the degradation properties of strain YYML68 against ι-, κ- and λ-carrageenan. From this degradation test, we observed a significant 1.5-fold relative reduction in culture media viscosity at 24 h only when strain YYML68 was incubated with ι-carrageenan (Figure 2). In contrast, no reduction in media viscosity was observed when incubated with κ- and λ-carrageenan. Our results attained from the preliminary genome analysis and degradation test suggest that strain YYML68 harbors functional ι-carrageenases, and that *cgi*A and *cgi*B may be the candidate genes encoding for the enzyme.

### 3.2. CPP–PNA Efficiently Suppresses Protein Translation in Strain YYML68

**Figure 3:**
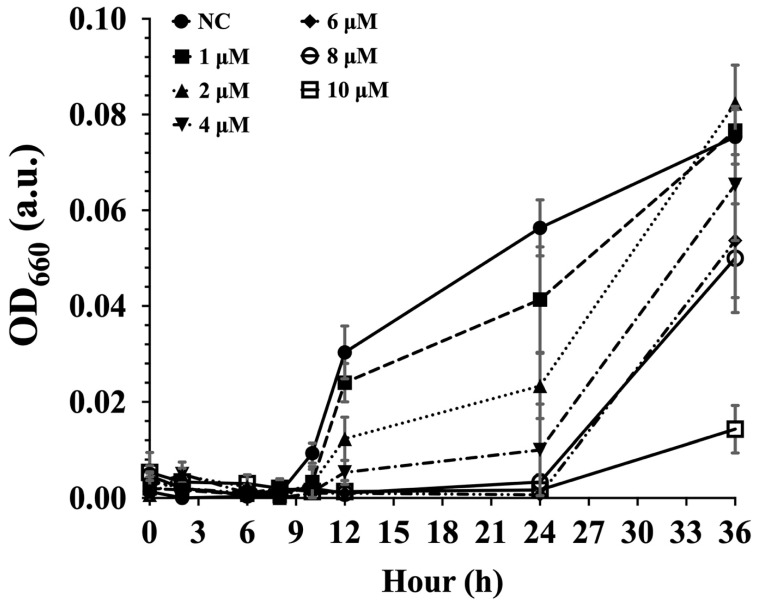
Growth inhibition analysis of *Paenibacillus* sp. YYML68. Strain YYML68 were incubated with the inhibition probe, CPP-*Pae*68acp, at the designated concentrations (1–10 µM) in liquid culture under constant agitation at 30℃ for 36 h. Untreated YYML8 strain samples were used as a negative control (NC). Growth of strain YYML68 was determined based on optical density measured at 660 nm.

In our attempt to use CPP–PNA as a simple preliminary approach in protein function elucidation, we first evaluated the ability of CPP–PNA to regulate protein translation in strain YYML68. An *acp*P gene encoding for the acyl carrier protein, AcpP, was identified from the sequenced genome and was used as a target. The gene sequence of the AcpP protein was also registered at DDBJ/EMBL/GenBank under the accession number LC730802. We showed that the growth inhibition of strain YYML68 correlated with the concentration of CPP-*Pae*68acp probes used (Figure 3). The negative control showed an increase in cell growth to an OD_660_ of approximately 0.08, which was close to the final OD_660_ of a 2-day culture of approximately 0.1. However, with cultures treated with the CPP-*Pae*68acp probe, we observed a gradual reduction in cell growth with probe concentrations as low as 1 µM. Complete growth inhibition of strain YYML68 at 24 h was attained in cultures treated with 6 µM of CPP-*Pae*68acp probes and above. However, cell growth recovery was observed at 36 h, suggesting that the CPP-*Pae*68acp probe was non-toxic to strain YYML68.

**Figure 4:**
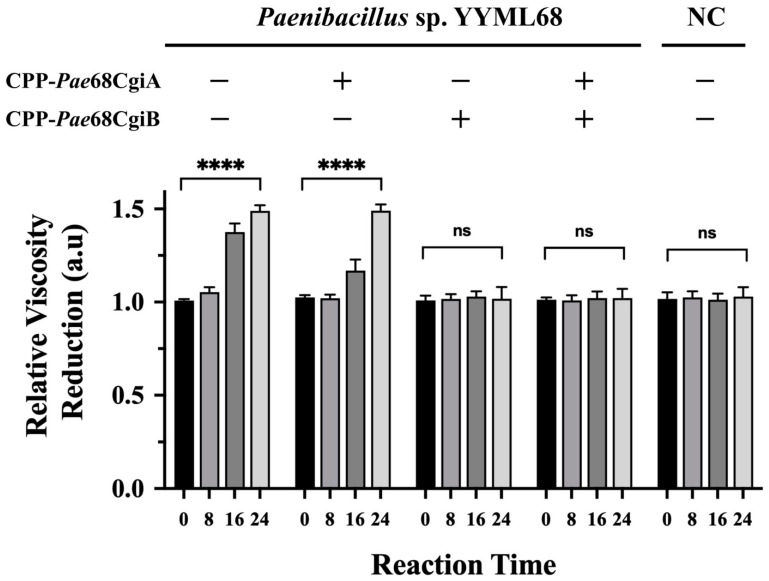
Functional analysis of the ι-carrageenase candidate genes, *cgi*A and *cgi*B, via CPP-PNA regulation. The concentration of ι-carrageenan and the designated probes was 1% and 8 µM, respectively. Samples were measured at 0, 8, 16 and 24 h time points, and degradation was determined by the reduction of media viscosity in comparison to a non-carrageenan degrading bacterium, *Bacillus megateirum* (Negative control; NC). All samples were performed in triplicates. Statistical significance (*p* < 0.05) was determined by *t*-test where **** indicates the level of significance. ns: non-significant.

Having shown that CPP-PNA may regulate protein translation in strain YYML68, we verified their effects against the ι-carrageenase candidate genes *cgi*A and *cgi*B, by treating strain YYML68 with either the CPP-*Pae*68cgiA or the CPP-*Pae*68cgiB probe, or with both the probes simultaneously. Using the similar carrageenan degradation test as before, cells treated only with the CPP-*Pae*68cgiA showed an approximate 1.5-fold relative reduction in culture media viscosity, similar to the cells not treated with either of the probes (Figure 4). On the other hand, we did not observe any significant reduction in culture media viscosity when strain YYML68 was treated with CPP-*Pae*68cgiB, when both of the probes were applied or with the negative control, where we used a non-carrageenan degrading bacterium, *B. megaterium*. To further justify the degradation of ι-carrageenan during this degradation test, the growth inhibition effects of each of the probes was evaluated and the degradation products of each sample were also analyzed using MALDI-TOF MS. From the growth inhibition analysis, neither of the probes showed any growth inhibition effects to strain YYML68 (Figure S3). From the MALDI-TOF MS analysis, three identical and distinct peaks of compounds, showing predicted and calculated molecular sizes equivalent to the oligomers, 3,6-anhydro-d-galactose-2-sulfate (DA2S) and d-galactose-4-sulfate (G4S) of ι-carrageenan, were observed from the samples treated with strain YYML68 only and samples treated with strain YYML68 in the presence of the CPP-*Pae*68cgiA probe (Figure S4)**.**

Based on these results, we conclude that there is a high possibility that the candidate gene *cgi*B encodes for a functional extracellularly secreted ι-carrageenase. Candidate gene *cgi*A, however, did not show significant ι-carrageenan degradation in the presence of the CPP-*Pae*68cgiB probe and, therefore, was regarded as not expressed or non-functional.

### 3.3. Carrageenase Gene Comparison

Showing that CPP-PNA was applicable in identifying the functional ι-carrageenase encoded by the *cgi*B gene, we took one step further to investigate the structure of the protein CgiB to see if it harbored unique features in comparison to currently known ι-carrageenases. Here, we also evaluated the structure of the CgiA protein to see if there were any significant hints to explain why the protein may be non-functional. For tertiary structure comparison, the protein sequence of CgiA and CgiB were uploaded to the SWISS-MODEL online 3D modelling server, and the results showed that the predicted structure of both proteins showed high similarity to the ι-carrageenase of *Alteromonas fortis* [29], with amino acid sequence coverage of 78% and 71%, and identities of 20.38% and 20.82%, respectively. As observed, both CgiA and CgiB were clearly missing domain A, a region known to be crucial for the formation of the enzyme catalytic tunnel required for ι-carrageenan degradation [30] among ι-carrageenases. Using the predicted tertiary structural conformation of the β-sheets and α-helixes of these proteins, they were subsequently aligned and compared at the secondary structure level with reported ι-carrageenases that were also reported to be missing domain A, including those from *M. thermotolerans* JAMB-A94 and *P. atlantica* T6c [27].

**Figure 5:**
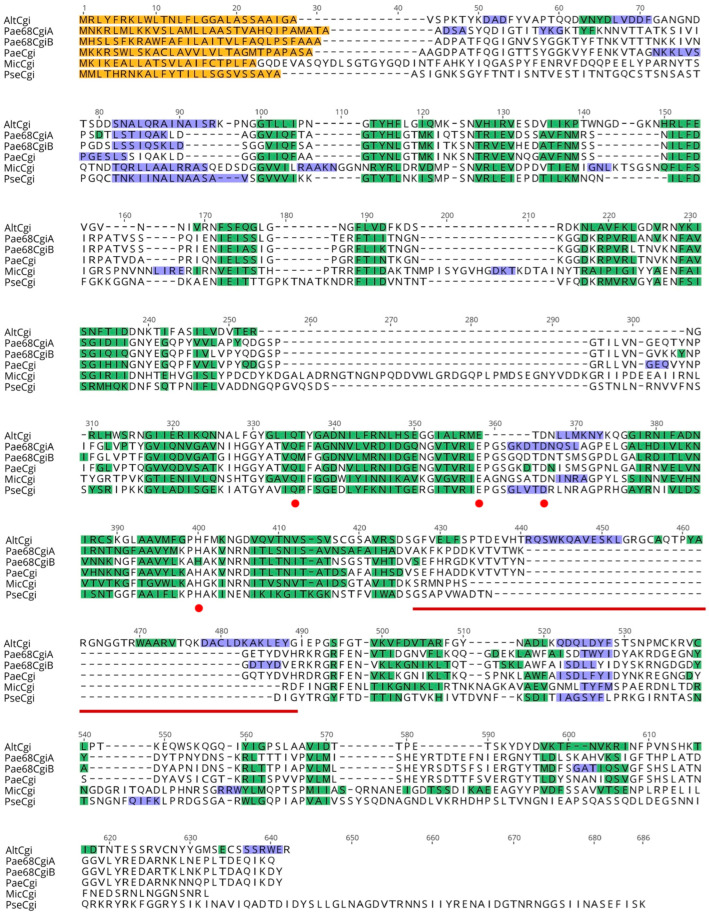
Multiple amino acid sequence alignment of the ι-carrageenase candidate proteins CgiA (Pae68CgiA) and CgiB (Pae68CgiB) of *Paenibacillus* strain YYML68 in comparison to reported ι-carrageenases. The ι-carrageenases from *Paenibacillus* sp. HB172198 (PaeCgi), *Microbulbifer thermotolerans* JAMB-A94 (MicCgi), *Pseudoalteromonas atlantica* T6c (PseCgi) and *Alteromonas fortis* (AltCgi) were used as the reference sequence. Amino acid residues highlighted in green and purple indicate ß-sheets and α-helix attained from predicted 3D models constructed using SWISS-MODEL. The red underline indicates domain A, and red dots indicate the important residues required for carrageenan degradation, as presented by Martin et al. [27].

The secondary structure comparison analysis was used to identify the important amino acid residues for ι-carrageenan degradation (Figure 5). Comparing CgiA and CgiB from strain YYML68 with the ι-carrageenase from another *Paenibacillus* strain HB172198, (isolated from brown agar, China, Hainan province) listed within the GH82 family of the CAZy database, we found that all three sequences were strikingly similar when multiple aligned. The sequences averaged at 480 amino acid residues in length with no prominent gaps; they share 296 identical sites (61.9%), and have a high pairwise identity of 71.0%. CgiA and CgiB also showed high structural similarity to the ι-carrageenases of *M. thermotolerans* JAMB-A94^T^ and *P. atlantica* T6c, and all the catalytic sites related to carrageenan degradation were highly conserved among all the proteins, including those identified from *A. fortis*.

## 4. Discussion

CPPs are short peptides that have properties to act as a carrier to deliver biomolecules into diverse prokaryotic and eukaryotic cells. In bacteria, CPPs have been employed for the delivery of numerous biomolecules, including nucleic acid, small compounds and proteins [31,32,33]. PNAs, on the other hand, are nucleotide analogues with an artificial peptide backbone comprising N-2-aminoethylglycine repeats that show strong binding affinity to DNA and RNA and resistance to intracellular enzymes including nucleases and proteases [34,35,36,37]. Due to the unique features of both these molecules, together, CPP-PNA conjugates can serve as probes to bind to specific nucleic acid sequences in live cells, namely with mRNA in vivo, and to inhibit the translation of proteins. CPP-PNA conjugates are currently employed in the biomedical field where growth inhibition of pathogenic strains or antibiotic-resistant bacteria have been reported [38,39,40]. Although showing potential application, as far as we know, CPP-PNA conjugates are still not fully exploited.

One possible major setback to the application of CPPs with bacteria is the limitation in its versatility. Reports thus far have shown that CPPs are species specific [41]. As such, more versatile CPPs applicable with diverse bacterial strains, particularly with novel or hard-to-transform strains, are required. Using several newly found properties of CPPs attained from our previous efforts, we employed our optimized CPPs with a hard-to-transform bacterium, *Paenibacillus* sp. strain YYML68. Genus *Paenibacillus* is known to produce numerous secondary metabolites important to industry, but its genetic manipulation has been limited to several strains, and reports use namely electroporation [42,43,44] or conjugation [45,46]. In this work, we used the (DabFF)_3_Dab CPP where we showed that this CPP could efficiently and easily permeate the membrane of *Paenibacillus* sp. strain YYML68 (Figure S5). Subsequently, upon designing a PNA targeting the mRNA of the acyl carrier protein, AcpP, and conjugating it to our CPP, we showed that CPP-PNA could arrest bacterial growth in a concentration dependent manner within a 24 h window upon CPP-PNA application. In addition, we also verified that CPP was not toxic to strain YYML68 where gradual growth recovery was observed for all probe concentrations at 36 h (Figure 3). Full growth recovery for the cells treated with 10 µM of the CPP-*Pae*68acp probes (OD_660_ equivalent to the non-treated cells) was observed at 96 h. Finally, we employed CPP-PNA as a tool to identify and elucidate the function of uncharacterized carrageenases within strain YYML68 (Figure 4).

Based on our tertiary and secondary structure comparative analysis of CgiA and CgiB with other ι-carrageenases, the most prominent feature of both CgiA and CgiB from strain YYML68 was the missing of domain A. However, our most interesting discovery was the inactivity of CgiA. Although CgiA harbored all the conserved catalytic sites discussed to be crucial in ι-carrageenan degradation (Figure 5), it did not show any activity when using the CPP-*Pae*68cgiB probe (Figure 4). Based on this analysis, we hypothesized two possible explanations to this phenomenon. The first would be the possibility of CgiA being expressed but not functional. Having performed the comparative analysis between the currently reported Clade C ι-carrageenases (Figure S1 and Figure 5), we showed that both proteins were highly similar in structure and have near identical catalytic sites. Therefore, a sound assumption to this difference could be due to the presence of other undiscovered catalytic sites. The second would be the possibility of strain YYML68 switching between the expression and utilization of CgiA and CgiB. Such an ability enhances the host to efficiently degrade in addition to producing different degraded isoforms of the target polysaccharide. However, showing here that CgiA and CgiB have near sequence and structural similarity, it is possible to assume that strain YYML68 may have a mechanism that controls the expression of both enzymes. As our current objective in this work was to show the applicability of CPP-PNA in in vivo protein functional analysis, further detailed study of these hypotheses was not pursued.

In this work, we showed the potentiality and applicability of using CPP-PNA as a tool for the identification and elucidation of uncharacterized proteins within a hard-to-transform bacterium. We showed that the method is simple since CPP–PNA probes designed to target the gene-of-interest are incubated with the target bacterium and can be assayed for several hours upon CPP-PNA incubation without the need to generate or select mutant strains. CPP-PNA is also advantageous, since gene regulation can be performed in a concentration-dependent manner, allowing it to be employed to elucidate and evaluate the function of enzymes within metabolic pathways that may cause cell death if their genes are completely knocked out.

However, although we clearly see the applicability of using CPP-PNA conjugates in protein functional analyses, several improvements still need to be considered. This includes the possibility of off-target effects that may occur due to the length of the PNA sequences used in this work which is only 10-mer. 10-mer was used as the length of our PNA, since it has been proven to permeate the cells at high efficiency and is sufficient to regulate genes as previously reported [47,48]. In similar specific sequence binding experiments using DNA, longer probes are favorable (approximately 20–30-mers) to increase binding specificity, but it is currently technically challenging to synthesize PNAs that are more than 15-mers in length [49]. Using standard peptide solid-phase synthesis protocols, we have been successful in synthesizing PNA probes to as long as 15-mers, but based on our experience and also from previously reported [47], CPP harboring PNA conjugates longer than 12-mer greatly affect the permeation efficiency of the conjugates into live cells. Methods to synthesize longer PNA sequences have been introduced [50,51], but for these probes to be applied, CPPs with the ability to deliver longer PNA into bacteria also need to be designed and evaluated. Based on the difference between the thermostability and affinity of PNAs binding to mRNA, which is more stable than DNA-RNA interactions, there is also the possibility of increase in non-specific partial binding of longer PNA probes to non-target mRNA molecules, hence, resulting in the possibility of increased off-target effects. Therefore, the length of PNA and its binding properties with DNA and its relativity to protein translation regulation should be further evaluated and optimized. Another potential challenge we see in using CPP-PNA conjugates for the characterization of novel proteins in vivo is that, similar to silencing RNA (siRNA), in contrast to knock-out mutant strains where gene expression of the target gene has been nullified and functional evaluation of the target gene can be performed immediately, the effects of CPP–PNA inhibition is only seen once the intracellular abundance of the target protein is lowered or depleted. Depletion of the target protein varies due to the expression of the protein ranging from several hours to days, and in most cases is best monitored using specific antibodies via western blotting or by assays that directly measure the reaction of the target protein. Thus, currently, CPP-PNA conjugates are more likely to be more applicable in evaluating or elucidating the function of proteins in which antibodies are available or where assays have been developed.

Despite some of the setbacks highlighted above, we believe that CPP-PNA conjugates have the potential to serve as a new approach to elucidate and characterize proteins in vivo within the native bacterium.

## Data Availability

The data sets including genome and gene sequences have been uploaded to the designated database and are linked to accession numbers that have been included in the main text.

## Supplementary Materials

The following are available online at https://www.mdpi.com/article/10.3390/molecules27248944/s1, Figure S1: Degradation of crude carrageenan by *Paenibacillus* sp. YYML68, Figure S2: Phylogenetic representation of the CgiA and CgiB candidate hypothetical proteins with the proteins of the GH82 family from the CAZy database, Figure S3: Growth inhibition effects of the CPP-*Pae*68CgiA and CPP-*Pae*68CgiB probes, Figure S4: MALDI-TOF MS analysis of the degradation products by CgiA and CgiB, Figure S5: Evaluation of (DabFF)_3_K-FAM permeation into *Paenibacillus* sp. YYML68. References from Supplementary materials [52,53,54].

## References

[1] Brown T.A. (2021). Gene Cloning and DNA Analysis: An Introduction.

[2] Terpe K. (2006). Overview of bacterial expression systems for heterologous protein production: From molecular and biochemical fundamentals to commercial systems. Appl. Microbiol. Biotechnol..

[3] Freudl R. (2018). Signal peptides for recombinant protein secretion in bacterial expression systems. Microb. Cell Factories.

[4] Silverman A.D., Karim A.S., Jewett M.C. (2020). Cell-free gene expression: An expanded repertoire of applications. Nat. Rev. Genet..

[5] Tripathi N.K., Shrivastava A. (2019). Recent Developments in Bioprocessing of Recombinant Proteins: Expression Hosts and Process Development. Front. Bioeng. Biotechnol..

[6] Souque C., Escudero J.A., MacLean R.C. (2021). Integron activity accelerates the evolution of antibiotic resistance. Elife.

[7] Desler C., Durhuus J.A., Rasmussen L.J. (2012). Genome-wide screens for expressed hypothetical proteins. Methods Mol. Biol..

[8] Sharan R., Ulitsky I., Shamir R. (2007). Network-based prediction of protein function. Mol. Syst. Biol..

[9] Becker S.A., Palsson B.O. (2008). Three factors underlying incorrect in silico predictions of essential metabolic genes. BMC Syst Biol.

[10] Jones C.E., Brown A.L., Baumann U. (2007). Estimating the annotation error rate of curated GO database sequence annotations. BMC Bioinform..

[11] Schnoes A.M., Brown S.D., Dodevski I., Babbitt P.C. (2009). Annotation error in public databases: Misannotation of molecular function in enzyme superfamilies. PLoS Comput. Biol..

[12] Rembeza E., Engqvist M.K.M. (2021). Experimental and computational investigation of enzyme functional annotations uncovers misannotation in the EC 1.1.3.15 enzyme class. PLoS Comput. Biol..

[13] Chen X., Li C., Liu H. (2021). Enhanced Recombinant Protein Production Under Special Environmental Stress. Front. Microbiol..

[14] Li L., Maclntyre L.W., Brady S.F. (2021). Refactoring biosynthetic gene clusters for heterologous production of microbial natural products. Curr. Opin. Biotechnol..

[15] Bauman K.D., Li J., Murata K., Mantovani S.M., Dahesh S., Nizet V., Luhavaya H., Moore B.S. (2019). Refactoring the Cryptic Streptophenazine Biosynthetic Gene Cluster Unites Phenazine, Polyketide, and Nonribosomal Peptide Biochemistry. Cell Chem. Biol..

[16] Zhang X., Hindra, Elliot M.A. (2019). Unlocking the trove of metabolic treasures: Activating silent biosynthetic gene clusters in bacteria and fungi. Curr. Opin. Microbiol..

[17] Toyohara D., Yokoi Y., Inoue G., Muraoka T., Mori T. (2019). Abiotic Factors Promote Cell Penetrating Peptide Permeability in Enterobacteriaceae Models. Front. Microbiol..

[18] Inoue G., Toyohara D., Mori T., Muraoka T. (2021). Critical Side Chain Effects of Cell-Penetrating Peptides for Transporting Oligo Peptide Nucleic Acids in Bacteria. ACS Appl. Biol. Mater..

[19] De Coster W., D’Hert S., Schultz D.T., Cruts M., Van Broeckhoven C. (2018). NanoPack: Visualizing and processing long-read sequencing data. Bioinformatics.

[20] Kolmogorov M., Yuan J., Lin Y., Pevzner P.A. (2019). Assembly of long, error-prone reads using repeat graphs. Nat. Biotechnol..

[21] Walker B.J., Abeel T., Shea T., Priest M., Abouelliel A., Sakthikumar S., Cuomo C.A., Zeng Q., Wortman J., Young S.K. (2014). Pilon: An integrated tool for comprehensive microbial variant detection and genome assembly improvement. PLoS ONE.

[22] Bolger A.M., Lohse M., Usadel B. (2014). Trimmomatic: A flexible trimmer for Illumina sequence data. Bioinformatics.

[23] Simao F.A., Waterhouse R.M., Ioannidis P., Kriventseva E.V., Zdobnov E.M. (2015). BUSCO: Assessing genome assembly and annotation completeness with single-copy orthologs. Bioinformatics.

[24] Brettin T., Davis J.J., Disz T., Edwards R.A., Gerdes S., Olsen G.J., Olson R., Overbeek R., Parrello B., Pusch G.D. (2015). RASTtk: A modular and extensible implementation of the RAST algorithm for building custom annotation pipelines and annotating batches of genomes. Sci. Rep..

[25] Zhang H., Yohe T., Huang L., Entwistle S., Wu P., Yang Z., Busk P.K., Xu Y., Yin Y. (2018). dbCAN2: A meta server for automated carbohydrate-active enzyme annotation. Nucleic Acids Res..

[26] Waterhouse A., Bertoni M., Bienert S., Studer G., Tauriello G., Gumienny R., Heer F.T., de Beer T.A.P., Rempfer C., Bordoli L. (2018). SWISS-MODEL: Homology modelling of protein structures and complexes. Nucleic Acids Res..

[27] Martin M., Vandermies M., Joyeux C., Martin R., Barbeyron T., Michel G., Vandenbol M. (2016). Discovering novel enzymes by functional screening of plurigenomic libraries from alga-associated Flavobacteriia and Gammaproteobacteria. Microbiol. Res..

[28] Hatada Y., Mizuno M., Li Z., Ohta Y. (2011). Hyper-production and characterization of the iota-carrageenase useful for iota-carrageenan oligosaccharide production from a deep-sea bacterium, Microbulbifer thermotolerans JAMB-A94T, and insight into the unusual catalytic mechanism. Mar. Biotechnol..

[29] Michel G., Chantalat L., Fanchon E., Henrissat B., Kloareg B., Dideberg O. (2001). The iota-carrageenase of Alteromonas fortis. A beta-helix fold-containing enzyme for the degradation of a highly polyanionic polysaccharide. J. Biol. Chem..

[30] Michel G., Helbert W., Kahn R., Dideberg O., Kloareg B. (2003). The structural bases of the processive degradation of iota-carrageenan, a main cell wall polysaccharide of red algae. J. Mol. Biol..

[31] Hejtmankova A., Vanova J., Spanielova H. (2021). Cell-penetrating peptides in the intracellular delivery of viral nanoparticles. Vitam. Horm..

[32] Palm-Apergi C., Dowdy S.F. (2022). Protein Delivery by PTDs/CPPs. Methods Mol. Biol..

[33] Ruter C. (2022). Delivery of Antibiotics by Cell-Penetrating Peptides to Kill Intracellular Pathogens. Methods Mol. Biol..

[34] Perera J.D.R., Carufe K.E.W., Glazer P.M. (2021). Peptide nucleic acids and their role in gene regulation and editing. Biopolymers.

[35] Sun H., Kong J., Zhang X. (2021). Application of peptide nucleic acid in electrochemical nucleic acid biosensors. Biopolymers.

[36] Nacher-Vazquez M., Santos B., Azevedo N.F., Cerqueira L. (2022). The role of Nucleic Acid Mimics (NAMs) on FISH-based techniques and applications for microbial detection. Microbiol. Res..

[37] Gupta A., Mishra A., Puri N. (2017). Peptide nucleic acids: Advanced tools for biomedical applications. J. Biotechnol..

[38] Narenji H., Teymournejad O., Rezaee M.A., Taghizadeh S., Mehramuz B., Aghazadeh M., Asgharzadeh M., Madhi M., Gholizadeh P., Ganbarov K. (2020). Antisense peptide nucleic acids againstftsZ andefaA genes inhibit growth and biofilm formation of Enterococcusfaecalis. Microb. Pathog..

[39] Javanmard Z., Kalani B.S., Razavi S., Farahani N.N., Mohammadzadeh R., Javanmard F., Irajian G. (2020). Evaluation of cell-penetrating peptide-peptide nucleic acid effect in the inhibition of cagA in Helicobacter pylori. Acta Microbiol. Immunol. Hung..

[40] da Silva K.E., Ribeiro S.M., Rossato L., Dos Santos C.P., Preza S.E., Cardoso M.H., Franco O.L., Migliolo L., Simionatto S. (2021). Antisense peptide nucleic acid inhibits the growth of KPC-producing Klebsiella pneumoniae strain. Res. Microbiol..

[41] Xue X.Y., Mao X.G., Zhou Y., Chen Z., Hu Y., Hou Z., Li M.K., Meng J.R., Luo X.X. (2018). Advances in the delivery of antisense oligonucleotides for combating bacterial infectious diseases. Nanomedicine.

[42] Okonkwo C.C., Ujor V., Cornish K., Ezeji T.C. (2020). Inactivation of the Levansucrase Gene in Paenibacillus polymyxa DSM 365 Diminishes Exopolysaccharide Biosynthesis during 2,3-Butanediol Fermentation. Appl. Environ. Microbiol..

[43] Descamps T., De Smet L., De Vos P., de Graaf D.C. (2018). Unbiased random mutagenesis contributes to a better understanding of the virulent behaviour of Paenibacillus larvae. J. Appl. Microbiol..

[44] Murray K.D., Aronstein K.A. (2008). Transformation of the Gram-positive honey bee pathogen, Paenibacillus larvae, by electroporation. J. Microbiol. Methods.

[45] Heinze S., Kornberger P., Gratz C., Schwarz W.H., Zverlov V.V., Liebl W. (2018). Transmating: Conjugative transfer of a new broad host range expression vector to various Bacillus species using a single protocol. BMC Microbiol..

[46] Meliawati M., May T., Eckerlin J., Heinrich D., Herold A., Schmid J. (2022). Insights in the Complex DegU, DegS, and Spo0A Regulation System of Paenibacillus polymyxa by CRISPR-Cas9-Based Targeted Point Mutations. Appl. Environ. Microbiol..

[47] Good L., Awasthi S.K., Dryselius R., Larsson O., Nielsen P.E. (2001). Bactericidal antisense effects of peptide-PNA conjugates. Nat. Biotechnol..

[48] Patel R.R., Sundin G.W., Yang C.H., Wang J., Huntley R.B., Yuan X., Zeng Q. (2017). Exploration of Using Antisense Peptide Nucleic Acid (PNA)-cell Penetrating Peptide (CPP) as a Novel Bactericide against Fire Blight Pathogen Erwinia amylovora. Front. Microbiol..

[49] Tailhades J., Takizawa H., Gait M.J., Wellings D.A., Wade J.D., Aoki Y., Shabanpoor F. (2017). Solid-Phase Synthesis of Difficult Purine-Rich PNAs through Selective Hmb Incorporation: Application to the Total Synthesis of Cell Penetrating Peptide-PNAs. Front. Chem..

[50] Patil N.A., Thombare V.J., Li R., He X., Lu J., Yu H.H., Wickremasinghe H., Pamulapati K., Azad M.A.K., Velkov T. (2022). An Efficient Approach for the Design and Synthesis of Antimicrobial Peptide-Peptide Nucleic Acid Conjugates. Front. Chem..

[51] Li C., Callahan A.J., Phadke K.S., Bellaire B., Farquhar C.E., Zhang G., Schissel C.K., Mijalis A.J., Hartrampf N., Loas A. (2022). Automated Flow Synthesis of Peptide-PNA Conjugates. ACS Cent. Sci..

[52] Michel G., Czjzek M., Trincone A. (2013). Polysaccharide-degrading enzymes from marine bacteria. Marine Enzymes for Biocatalysis: Sources, Biocatalytic Characteristic and Bioprocesses of Marine Enzymes.

[53] Antonopoulos A., Favetta P., Helbert W., Lafosse M. (2005). On-line liquid chromatography electrospray ionization mass spectrometry for the characterization of kappa- and iota-carrageenans. Application to the hybrid iota-/nu-carrageenans. Anal. Chem..

[54] Fatema M.K., Nonami H., Ducatti D.R., Goncalves A.G., Duarte M.E., Noseda M.D., Cerezo A.S., Erra-Balsells R., Matulewicz M.C. (2010). Matrix-assisted laser desorption/ionization time-of-flight (MALDI-TOF) mass spectrometry analysis of oligosaccharides and oligosaccharide alditols obtained by hydrolysis of agaroses and carrageenans, two important types of red seaweed polysaccharides. Carbohydr. Res..

