# Supplementary material for: Cell-Penetrating Peptide–Peptide Nucleic Acid Conjugates as a Tool for Protein Functional Elucidation in the Native Bacterium
Source: Molecules. 2022 Dec 15;27(24):8944. doi: 10.3390/molecules27248944 (PMC9788395; doi:10.3390/molecules27248944)
Supplement: Supplementary file 1 [file molecules-27-08944-s001.zip › molecules-1978292-supplementary.pdf]

Supplementary Material

Cell-Penetrating Peptide–Peptide Nucleic Acid Conjugates as a Tool for Protein Functional Elucidation in the Native Bacterium

Yasuhito Yokoi [1,†], Yugo Kawabuchi [1,†], Abdullah Adham Zulmajdi [1], Reiji Tanaka [2], Toshiyuki Shibata [2], Takahiro Muraoka [3] and Tetsushi Mori [1,*]

[1] Department of Biotechnology and Life Science, Tokyo University of Agriculture and Technology, 2-24-16 Naka-cho, Koganei-shi 184-8588, Tokyo, Japan
[2] Department of Life Sciences, Graduate School of Bioresources, Mie University, 1577 Kurima-machiya-cho, Tsu-shi 514-8507, Mie, Japan
[3] Department of Applied Chemistry, Graduate School of Engineering, Tokyo University of Agriculture and Technology, 2-24-16 Naka-cho, Koganei-shi 184-8588, Tokyo, Japan
* Correspondence: moritets@go.tuat.ac.jp
† These authors contributed equally to this work.

Citation: Yokoi, Y.; Kawabuchi, Y.; Zulmajdi, A.A.; Tanaka, R.; Shibata, T.; Muraoka, T.; Mori, T. Cell-Penetrating Peptide–Peptide Nucleic Acid Conjugates as a Tool for Protein Functional Elucidation in the Native Bacterium. *Molecules* **2022**, *27*, 8944. https://doi.org/10.3390/molecules27248944

Academic Editor: Paolo Iadarola

Received: 3 October 2022
Accepted: 12 December 2022
Published: 15 December 2022

Publisher's Note: MDPI stays neutral with regard to jurisdictional claims in published maps and institutional affiliations.

Copyright: © 2022 by the authors. Licensee MDPI, Basel, Switzerland. This article is an open access article distributed under the terms and conditions of the Creative Commons Attribution (CC BY) license (https://creativecommons.org/licenses/by/4.0/).

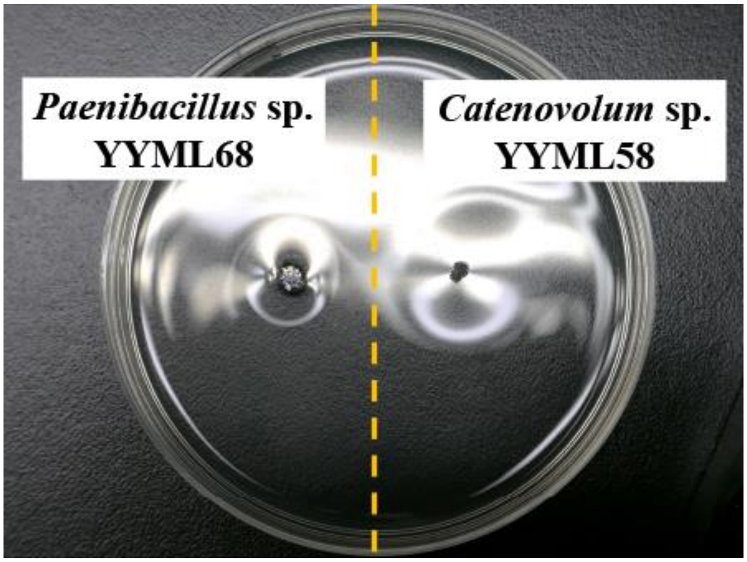

Figure S1. Degradation of crude carrageenan by *Paenibacillus* sp. YYML68. Strain YYML68 was cultivated on 4X diluted MB plates supplemented with 2% carrageenan for 2 days and carrageenan degradation was confirmed by “potholes” surrounding the bacterial colony. Another carrageenan-degrading isolate, *Catenovulum* sp. YYML58 was used as a control to indicate the efficiency of carrageenan degradation by strain YYML68.

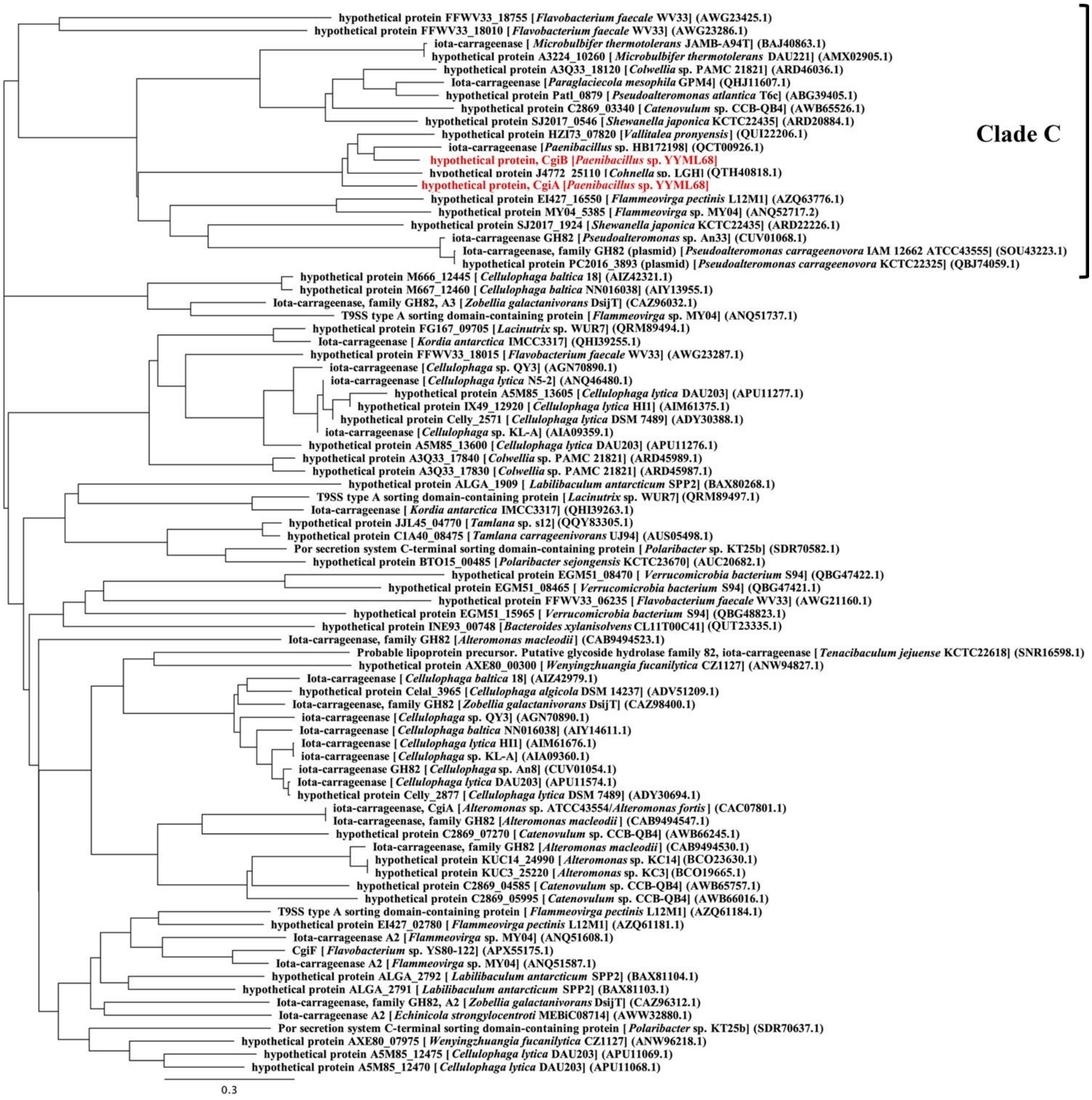

Figure S2. Phylogenetic representation of the CgiA and CgiB candidate hypothetical proteins with the proteins of the GH82 family from the CAZy database. The candidate genes are highlighted in red and were clustered within Clade C of the ι-carrageenase clades [1].

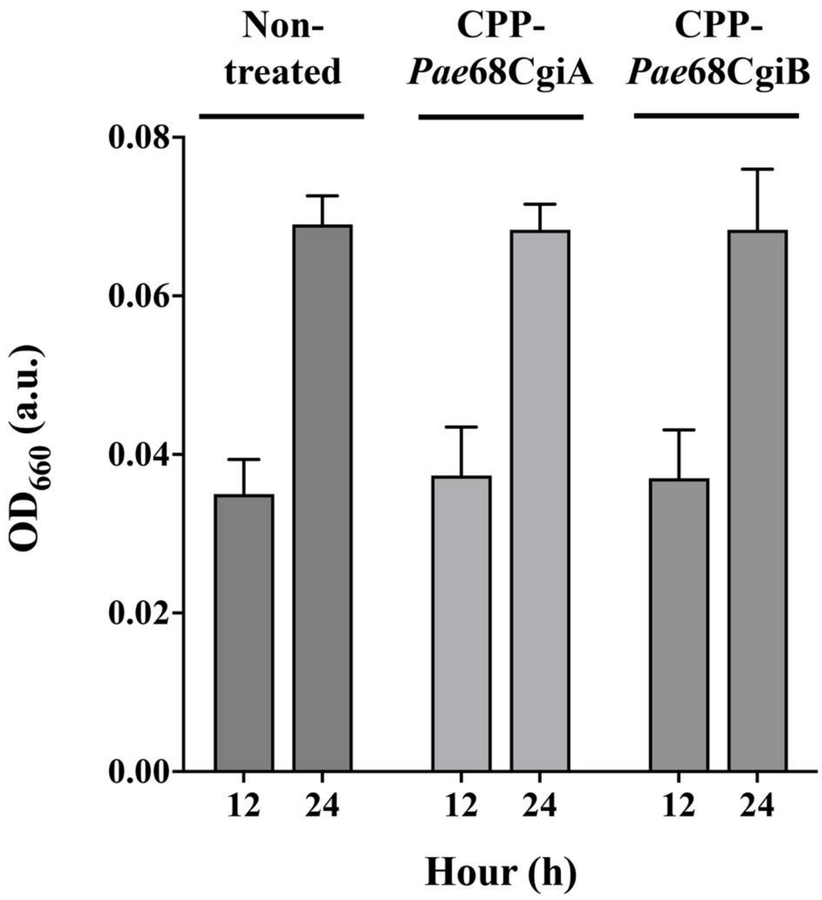

Figure S3. Growth inhibition effects of the CPP-*Pae*68CgiA and CPP-*Pae*68CgiB probes. 1.0 X 10^5 cells/mL were used as starter cultures and samples were cultivated in Marine Broth with the presence of the probes for 24 h. A non-probe treated samples was used as the negative control. The growth of the cells was measured at 12 and 24 h respectively.

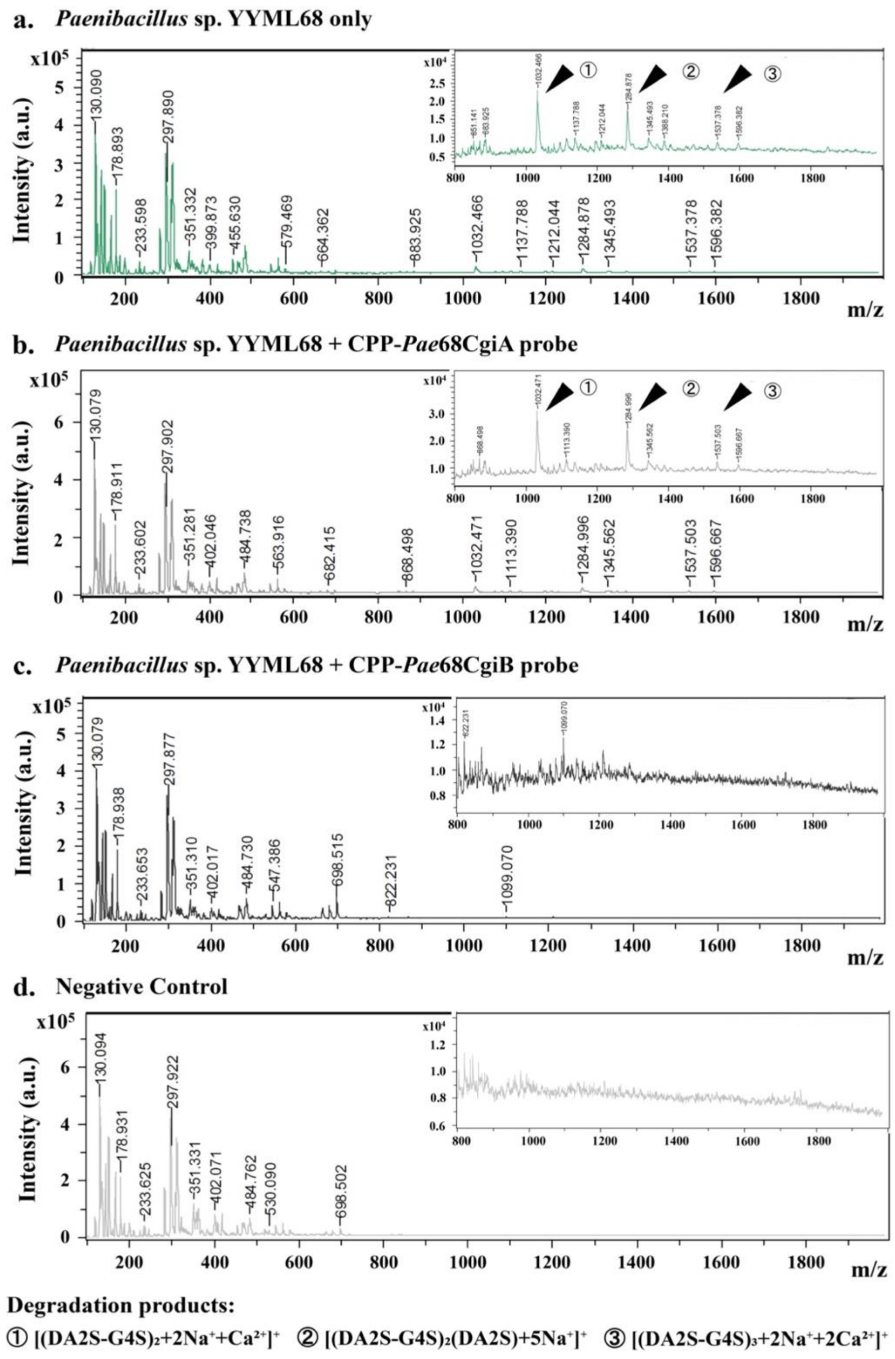

Figure S4. MALDI-TOF MS analysis of the degradation products by CgiA and CgiB. The measured m/z values of each peak averaged at ①1032.5 (1032.09 calculated), ②1284.9 (1285.15 calculated) and ③1537.4 (1536.2 calculated) respectively calculated based on the oligosaccharide values provided by Antonopoulos et al. [2]. Degradation experiments were performed in 0.01X Marine Broth (MB) supplemented with 0.5% ι-carrageenan with only the (a.) YYML68 strain, the (b.) YYML68 strain with the CPP-*Pae*CgiA probe, or the (c.) YYML68 strain with the CPP-*Pae*CgiB probe. A non-strain YYML68 inoculated sample was used as the (d.) negative control. MALDI-TOF MS analysis was performed using norharmane as the matrix [3] and measurements were performed at the positive ion mode.

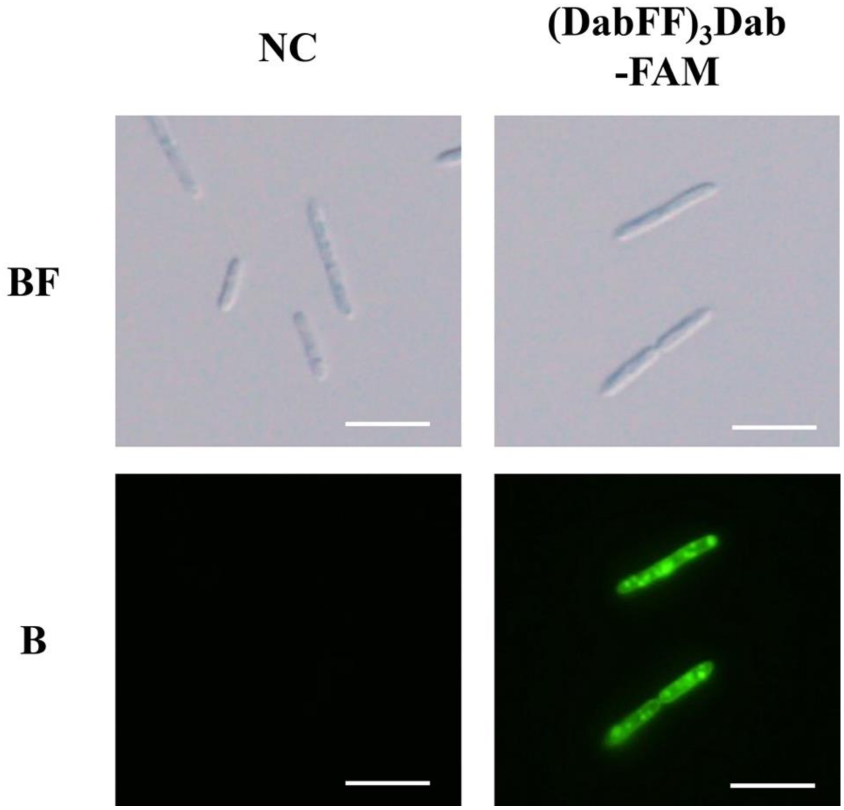

Figure S5. Evaluation of $(DabFF)_3$K-FAM permeation into *Paenibacillus* sp. YYML68. $(DabFF)_3$Dab-FAM *permeation* was performed at 2 µM concentration in PBS at room temperature (23°C) for 1 h. Untreated YYML8 strain samples were used as a negative control (NC). Green fluorescence images were captured using the blue wide-excitation mirror unit (U-FBWA; Olympus). Cell integrity was confirmed by bright field (BF) images. Images of $(DabFF)_3$Dab-FAM permeated cells and the negative controls were captured at similar exposure times to allow for comparison. Scale bar is 5 µm.

REFERENCES

1. Michel, G.; Czjzek, M., Polysaccharide-degrading enzymes from marine bacteria. In *Marine Enzymes for Biocatalysis: Sources, Biocatalytic Characteristic and Bioprocesses of Marine Enzymes*, Trincone, A., Ed. Woodhead Publishing Limited: Cambridge, 2013; pp 429-464.

2. Antonopoulos, A.; Favetta, P.; Helbert, W.; Lafosse, M., On-line liquid chromatography electrospray ionization mass spectrometry for the characterization of kappa- and iota-carrageenans. Application to the hybrid iota-/nu-carrageenans. *Analytical chemistry* **2005,** 77, (13), 4125-36.

3. Fatema, M. K.; Nonami, H.; Ducatti, D. R.; Goncalves, A. G.; Duarte, M. E.; Noseda, M. D.; Cerezo, A. S.; Erra-Balsells, R.; Matulewicz, M. C., Matrix-assisted laser desorption/ionization time-of-flight (MALDI-TOF) mass spectrometry analysis of oligosaccharides and oligosaccharide alditols obtained by hydrolysis of agaroses and carrageenans, two important types of red seaweed polysaccharides. *Carbohydrate research* **2010,** 345, (2), 275-83.
